# Will China-Africa trade increase Africa’s carbon emissions?

**DOI:** 10.1371/journal.pone.0289792

**Published:** 2023-11-17

**Authors:** Jiu-Jin Li, Jiemin Huang, Chen Wen, Shuang Zhang

**Affiliations:** 1 College of Economics and Management, Northeast Petroleum University, Daqing, China; 2 Shenzhen Institute of Information Technology, Shenzhen, Guangdong Province, China; 3 University of Electronic Science and Technology, Chengdu, China; University of Galway, Ireland / Anhui University of Finance and Economics, CHINA

## Abstract

In order to explore whether China-Africa exchange will influence on the African environment. This paper selects four paths of China-Africa exchanges and explores the impact of each path on the African environment under the influence of different factors. We found that construction income and Africa’s exports to China will increase Africa’s carbon emissions. Foreign direct investment and China’s exports to Africa will lead to a reduction in carbon emissions in Africa. The resource moderation will reduce the significance of the environmental impact of each path on Africa. Based on the above conclusions, several suggestions are made on the policies and actual operations in the path of China-Africa exchanges.

## 1 Introduction

The issue of global climate change has become an important topic for global society, governments and scientific community. There is a growing awareness of the impact of climate change on human societies and ecosystems. With the development of the UN sustainable development agenda, including the UN Sustainable Development Goals (SDGs), countries are beginning to focus on how to achieve environmental sustainability alongside economic development. The non-African continent is one of the most vulnerable regions in the world to climate change. Many African countries are challenged by climate change impacts such as extreme weather events, droughts, floods and sea level rise. This makes the need for African countries to reduce carbon emissions and adapt to climate change even more urgent. In recent years, trade and economic cooperation between China and African countries has been increasing. China is one of the continent’s largest trading partners, and trade between the two sides is increasing.According to the China Africa Research Initiative hosted by John Hopgkins University, Africa is China’s largest financial commitment outside Asia (http://www.sais-cari.org/data-china-africa-trade). Africa has abundant resources, a large population and potential markets, while China has accumulated rich experience and mature technologies in the fields of industry, agriculture and infrastructure construction. China’s sustainable development needs the support of resources and markets, while Africa has these advantages, thus China’s trade and investment in Africa is complementary.This has brought attention to the environmental impacts of China-Africa trade on the continent, including the increase in carbon emissions. Studying the relationship between China-Africa trade and carbon emissions in Africa can help to assess the progress and challenges of African countries in achieving the Sustainable Development Goals (SDGs). Such studies can also help to provide the scientific basis and policy recommendations to promote low-carbon development, climate change adaptation and achieve sustainable development.

Trade and investment between China and Africa will also have impact on African environment. According to World Development Indicators of 2014, China’s carbon dioxide emissions are 7.54 metric tons, one of the highest in the world (http://www.sais-cari.org/data-china-africa-trade). There are two competing theories about the impact of China on Africa’s environment: pollution haven hypothesis and pollution halo hypothesis. According to pollution haven hypothesis, pollution-intensive industries tend to be established in countries or regions with relatively low environmental standards. In contrast, the pollution halo hypothesis holds that foreign direct investment and trade improve environmental quality through technology transfer and the spread of advanced management ideas. China is in a period of rapid economic development and its influence on world economy is increasing. In order to maintain development, China may engage in activities to increase carbon emissions. Therefore, the pollution haven hypothesis may be embodied in some contact routes between Africa. On the contrary, China also has technology to ensure the efficient use of resources and has made achievements in the use of renewable energy. Therefore, consistent with the pollution halo hypothesis, China’s investment in Africa helps reduce carbon emissions in African countries, and African climate is more suitable for China to implement renewable energy technologies. Therefore, will China-Africa trade increase carbon emissions in Africa? If it will increase, what specific factors present in China-Africa trade will affect CO2 emissions is a question worth exploring. In the past, scholars’ research mostly focused on a single path, and focused more on the relationship between foreign direct investment or trade and the environment. Based on previous scholars’ research on the relationship between a single path and the environment [1], other paths that may have impact on the environment have been added. In this paper, agricultural and industrial factors are added to the environmental impacts of construction, exports, imports and foreign direct investment. China’s construction, exports, imports and FDI in Africa may increase Africa’s carbon emissions through transactions involving large-scale infrastructure development, manufacturing and the energy sector, where energy consumption and use of traditional high-carbon energy sources are more prominent. However, the Chinese government has taken a series of measures to address climate change and reduce carbon emissions, and has partnered with African countries to promote clean energy and low-carbon technologies. This paper examines the impact of construction, exports, imports and foreign direct investment on carbon emissions in Africa based on the above analysis.

## 2. Literature review

The existing literature on the relationship between international trade and environment offers two opposing perspectives on environmental effects of international trade: the pollution haven hypothesis and the pollution halo hypothesis. We have compiled the relevant theories of these two hypotheses.

### 2.1 Pollution haven hypothesis

Some scholars have studied the pollution haven hypothesis from the perspective of the exchanges between countries. Taking the data generated by trade between China and other Asian countries and developed countries as the research object, it examines the impact of trade between China and developed countries on environment [2]. The results show that China and other Asian countries have more pollution than developed countries, and China and other Asian countries have become pollution haven for the developed countries, which proves the pollution haven hypothesis. (The validity of the Côte d’Ivoire pollution haven hypothesis is studied by Assamoi et al [3].s The results prove that Côte d’Ivoire supports the pollution haven hypothesis because there is a positive relationship between foreign direct investment and CO2 emissions. Canberk & Suleyman [4] use the pollution haven hypothesis to investigate the impact of FDI on Korea’s environmental quality. The results show that the increase in FDI to South Korea has led to increase in per capita carbon dioxide emissions, supporting the pollution haven hypothesis. Shahbaz et al., [5] Essandoh et al.and [6] Umit et al. [7] found that FDI aggravated environmental degradation, which confirmed the pollution haven hypothesis. Akwasi et al.,[8]; Bulus et al. [9] and Destek et al. [10] conducted research in developed and developing countries and found that FDI inflows have a negative impact on host countries, proving the pollution haven hypothesis. Hao [11] conducted a study in both developed and developing countries and found that FDI inflows have a negative impact on the host country, proving the pollution haven hypothesis.

These studies provide an understanding of the pollution haven hypothesis, but the natural limitations of the studies make it difficult to determine causality and cannot completely rule out other potential explanations or complex relationships between variables. The time horizon of these studies may be limited, examining only specific time periods or specific economic environmental conditions. Thus, an integrated consideration of the effects over time and across economic cycles is lacking, and further research is needed to overcome potential limitations and shortcomings to ensure the accuracy and reliability of the findings.

### 2.2. Pollution halo hypothesis

Birdsall & Wheeler [12] first proposed the pollution halo hypothesis. Contrary to pollution haven hypothesis, they believed that trade and investment between developed and developing countries provided environmental protection technologies for developing countries, which was beneficial to the environment of developing countries. Later, many scholars proved the pollution halo hypothesis by studying exchanges between countries and between domestic regions. The study found that developing countries, when attracting foreign direct investment, found that enhanced audits and agreements with foreign investors on technology transfer could avoid environmental damage when accepting foreign direct investment [13]. Ren [14] and Wu [15] found that OFDI and Internet communication technologies can increase local green total factor productivity and reduce the environmental impact of CO2, but Wu [16] further states that green total factor productivity is related to the specific type of environmental regulation decentralization. The environmental pollutants carbon dioxide, sulfur dioxide, nitrogen oxides, etc. are the research objects. The results show that trade liberalization can reduce global carbon dioxide, sulfur dioxide and nitrogen oxide emissions, supporting the pollution halo hypothesis [17]. The validity of the pollution haven hypothesis in South Asian economies is tested. Research suggests that South Asian countries should focus on attracting clean foreign investment, while renewable energy production is critical to mitigating climate change [18]. (Salehnia et al. [19], Mehmet [20], and Danish et al. [21] found that the entry of FDI contributes to the reduction of environmental pollution in host countries.

Through the above studies, it is found that most scholars cited support the idea of the pollution halo hypothesis by examining the emission data of environmental pollutants, but there may be uncertainty in the actual situation of technology transfer. developed countries may protect their core technologies, restrict the transfer of technologies or transfer only the more mature and economically feasible technologies, while advanced technologies for environmental protection may not be fully transferred.

### 2.3. The composite effect argument

The composite effect argument is a compromise between the pollution haven hypothesis and the pollution halo hypothesis, which holds that the environmental impact of international trade needs to be determined according to different conditions. Liu et al. [22] study whether China’s development meets the hypothesis of pollution safe havens. The results show that although exports transfer carbon dioxide emissions from other economies to China, imports can also help avoid emissions from certain carbon-intensive industries in China. Different industries support different pollution hypotheses. (Contrary to the above views, Xie et al. [23] found that FDI could reduce carbon dioxide emissions in emerging economies under certain conditions and supported the pollution halo hypothesis in underdeveloped areas. Bokpin [24] found that the inflow of foreign direct investment has a negative impact on the environment, but at the same time, if there are institutions to monitor foreign investors, foreign direct investment can have positive impact on the environment. It is believed that by strengthening the effective control of pollution in some industries and narrowing the technological gap with developed countries, the pollution caused by the production of export products can be reduced [25]. Some scholars also believe that international trade has no impact on environment at all. Demena & Afesorgbor [26] found that the potential impact of foreign direct investment on emissions is close to zero. The results show that Chinese OFDI increases domestic environmental pollution by improving the size of the economy (scale effect) [27]. However, the reverse technology spillover effect from OFDI improves the domestic technology level (technology effect) and optimizes the domestic industrial structure (composition effect), thus reducing domestic environmental pollution.

To sum up, scholars have drawn different conclusions about the environmental impacts of trade and investment in developed and developing countries under different conditions.

### 2.4 Factors affecting China-Africa trade on carbon emissions

The environmental impact of foreign trade on a country can be decomposed into three effects: the scale effect, the technology effect and the composition effect. The scale effect refers to the fact that as foreign trade expands, the size of the market and economy increases, and the degree of environmental degradation increases. The technology effect refers to the technological progress (e.g., acquiring or learning new technologies through trade) that accompanies the growth of trade, including the increase in the level of production technology and the advancement of energy conservation and emission reduction technology, which can improve environmental quality with other conditions remaining unchanged. The composition effect refers to the fact that as the level of trade increases and the division of labor in the market becomes more refined and deeper, a country will focus on producing products in which it has a comparative advantage. Thus, the composition effect depends on the type of products with comparative advantage. If these products are high-energy and high-polluting, the composition effect may lead to environmental degradation; conversely, if low-energy and low-polluting products are produced, environmental conditions may continue to improve. Overall, the impact of foreign trade on the level of environmental pollution in a country or region depends on the combination of these three effects. The scale effect dominates at low levels of per capita income, while the technology effect dominates when per capita income levels are high. abdallah Abdul-Mumuni [28] finds the presence effect of foreign direct investment on carbon emissions in sub-Saharan Africa using a nonlinear panel study that examines the effect of urbanization and non-renewable energy consumption on carbon emissions. Hussain Muhammad Noshab [29] examined the impact of urbanization and non-renewable energy consumption on carbon emissions in Africa using data from a sample of 54 African Union countries from 1996 to 2019.Tsaurai Kunofiwa. [30] also examined the impact of financial development on carbon emissions in Africa.L Zhou [31] looks at the path to achieving carbon neutrality targets in South Africa and the policies, plans and measures to reduce emissions involving power, industry, transport, buildings, agriculture and forestry, and identifies the main difficulties and challenges faced. In summary, existing scholars have laid the foundation for the study of the impact of carbon emissions in Africa, and this paper further enriches the study of the impact of foreign trade on carbon emissions in Africa by considering the impact of social factors such as corruption index on carbon emissions in Africa on the basis of this study.

### 2.5 Review

First, with regard to the pollution paradise hypothesis, China, as the largest developing country in the process of development, may cause environmental degradation in Africa by importing natural resources from African countries. Instead, the pollution halo hypothesis holds that China, as a country with renewable energy and smart technologies, has the potential to transfer clean technologies to Africa through exports and foreign direct investment, which will have a positive impact on the African environment. There are different views on the two theories, and the main point of debate is whether international trade between China and Africa will increase Africa’s carbon emissions, which is the focus of this study, and this paper further examines the two hypotheses in detail.

Secondly, In the past, the research of summary scholars found that these studies have laid the foundation for the impact of trade factors on carbon emissions, but few scholars have studied the impact of trade between Africa on Africa’s carbon emissions, nor have they detailed the relevant factors affecting Africa’s carbon emissions. Thirdly, most of the scholars’ research focuses on a single path, focusing more on foreign direct investment or the relationship between trade and the environment. builds on previous scholars’ research on the relationship between single pathways and the environment, adding other pathways that may have an impact on the environment [1].

In this paper, agricultural and industrial factors are added to the environmental impact of construction, exports, imports and foreign direct investment, and the impact of related factors such as corruption indices on carbon emissions is additionally analyzed. It examines the environmental impact of trade and investment between China and Africa from multiple paths, compares the views of the pollution paradise hypothesis with the pollution halo hypothesis, and focuses on China’s role and responsibilities as a developing country. Finally, such research perspectives and methods can provide new insights and understandings, revise and expand the pollution paradise hypothesis and pollution halo hypothesis, and provide useful references for future environmental protection and sustainable development.

In the past, scholars’ research mostly focused on a single path, and focused more on the relationship between foreign direct investment or trade and environment. Based on previous scholars’ research on the relationship between a single path and the environment, other paths that may have impact on environment have been added [1]. In this paper, agricultural and industrial factors are added to the environmental impacts of construction, exports, imports and foreign direct investment. The environmental impacts of trade and investment between China and Africa are examined from multiple pathways, comparing the views of the pollution paradise hypothesis and the pollution halo hypothesis, and focusing on China’s role and responsibilities as a developing country. Such a research perspective and approach can provide new insights and understandings that can provide useful references for future environmental protection and sustainable development.

## 3. Research methods

### 3.1 research samples and data sources

This research is based on the China-Africa Research Initiative of Johns Hopkins University (http://www.sais-cari.org/data-china-africa-trade) and the World Bank databas (https://databank.worldbank.org/indicator/NY.GDP.MKTP.KD.ZG/1ff4a498/

Popular-Indicators#.). We have selected the data from 2003 to 2014 of African countries with basically complete data as the observed values, as shown in Table 1.

**Table 1:** Sample countries.

countries	
Algeria	Libya
Angola	Mauritius
Benin	Morocco
Botswana	Mozambique
Cabo Verde	Namibia
Cameroon	Niger
Congo, Dem. Rep.	Nigeria
Egypt, Arab Rep.	Senegal
Eritrea	Seychelles
Ethiopia	South Africa
Gabon	Tanzania
Gambia, The	Togo
Ghana	Tunisia
Kenya	Zambia
Lesotho	Zimbabwe

### 3.2 Variable selection

In Table 2, they’re the variables selected, and the reason for choosing them as below. The study can explore the following theoretical elaborations: resource exploitation effect: construction and FDI may introduce advanced technologies and equipment that increase the resource exploitation capacity of African countries. This may lead to higher utilization of resources and increased production, which in turn increases energy consumption and CO2 emissions. Trade effects: Exports and imports can lead to commodity flows between African countries and China, which can affect energy consumption and CO2 emissions. Exports may imply that African countries produce and process more commodities, while imports imply the consumption of more Chinese produced goods. These activities may affect energy use and the associated CO2 emissions. Technology transfer effects: FDI and export pathways may facilitate technology transfer, particularly in clean energy and environmental technologies. China, as a country with renewable energy and smart technologies, may transfer these clean technologies to African countries through exports and FDI, thus reducing CO2 emissions in African countries. Resource dependence effect: Africa’s total imports of goods and services from China may reflect the degree of dependence of African countries on Chinese resources. If African countries are overly dependent on China for resource supply, it may increase their CO2. This is because the collection, processing, and transportation of resources may involve energy consumption and emissions, and over dependence on imports may exacerbate the pressure on energy consumption and CO2 emissions. In this study, carbon dioxide emissions were chosen as the dependent variable to represent an important indicator of the African environment. Carbon dioxide is one of the major greenhouse gases and its emissions are closely related to climate change and environmental quality. By examining the relationship between China-Africa exchange pathways and CO2 emissions, the impact of China-Africa trade on the African environment can be revealed. The independent variables are the four paths of China-Africa exchange: construction, FDI, export and import. The construction pathway refers to the total annual revenue of Chinese companies’ construction projects in Africa. This represents the scale of Chinese infrastructure construction and investment in Africa. FDI refers to Chinese foreign direct investment in African countries and reflects the economic activities of Chinese firms in Africa. Exports and imports represent the total exports of goods and services from Africa to China and the total imports of goods and services from China by Africa, respectively. These independent variables represent the different directions and forms of China-Africa trade. The study will focus on the analysis of the mechanisms between these independent variables and CO2 emissions.

**Table 2:** Variable selection.

	variables	Variable definition
Dependent variables	CO2 emissions	CO2 emissions represent carbon dioxide emissions.
Independent variables	Construction	the total annual income of Chinese enterprises in Africa’s construction projects
	FDI	China’s foreign direct investment in African countries
	Export	African exports the total amount of goods and services to China.
	Import	the total amount of goods and services that Africa imports from China (hereinafter referred to as: Africa imports from China).
Control variables	Energy use	the oil equivalent kilogram per capita, which is used to control the impact of energy use
	GDP growth	control economic growth and development
	GDP Percapita	control economic growth and development
	Population	control the impact of population factors on the environment
	Industrial added value	industrial growth as a percentage of GDP
	Resource moderation	reflect the direct relationship between the abundance of natural resources and each path
	Corruption index	social factors to control the impact of corruption factors on environment.
	Agricultural added value	agricultural growth as a percentage of GDP.

In the mechanism analysis, the effects of control variables also need to be considered [1]. The study chose energy use, GDP growth, GDP per capita, and population as the basic control variables, and added industrial value added and agricultural value added to control for the environmental impacts of industry and agriculture. Energy use, which is energy consumption expressed in kilograms of oil equivalent per capita, was used to control for the impact of energy use. High energy use may be associated with higher CO2 emissions. GDP growth and GDP per capita are used to control the environmental impact of economic growth and development. Economic growth is usually accompanied by an increase in energy consumption and CO2 emissions, but if African countries are able to achieve cleaner and sustainable development, the negative environmental impacts may be reduced. Demographic factors were chosen as control variables because an increase in population size may lead to an increase in resource consumption and carbon dioxide emissions. By controlling for demographic factors, the environmental impact of non-African exchange pathways can be analyzed more accurately.

**Fig 1:**
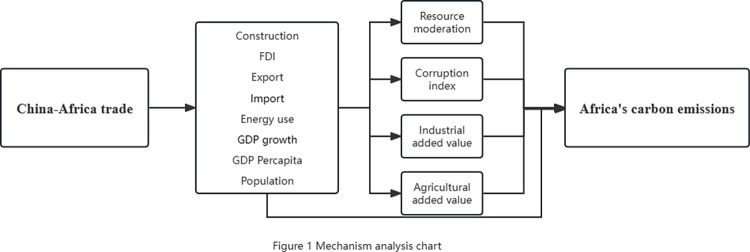
Mechanism analysis chart.

In addition, natural and social factors were introduced as control variables. The natural factor uses an indicator of resource conservation, which reflects the direct relationship between the abundance of natural resources and the pathways. This can help the study to better understand the relationship between resource use and CO2 emissions. Corruption index is used as a control variable for social factors to control the impact of corruption on the environment. Corruption can lead to waste of resources, non-compliance and poor environmental management, which in turn can have a negative impact on CO2 emissions. The mechanism is analyzed as shown below (Fig 1)

### 3.3 Model construction

By testing the panel data in this paper, it is found that the variable intercept model is suitable. The variable intercept model assumes that there is individual influence on the cross section but no structural change. The individual influence is expressed by the difference of intercept terms, that is, different individuals can have different intercept terms but the same coefficient [32]. The model is as follows:

$$y_{i}=\alpha_{i}+x_{i}\beta +\mu_{i},\;i=1,\;2,\;3,\;\ldots ,\;N$$ (1)

In the model: y_i_ is T×1 dimensional explained variable, x_i_ is T×k dimensional explanatory variable matrix, β is k×1 dimensional coefficient vector, intercept term between I section member equations α, i is different, which is used to reflect the influence of variables that reflect cross-section differences ignored in the model, and then the error term μ_i_ reflects the influence of section members and period change factors ignored in the model.

Because the four paths that affect the African environment are different, the following formulas are constructed (each path includes four formulas for resource moderation, corruption index, industrial added value and agricultural added value in the basic control variables):

(2) lnCO2_it_ = β_0_+β_1_Path_it_ +β_2_Control variables _it_+β_3_InEnergy use_it_+β_4_InGDP percapita_it_+β_5_InGDPgrowth_it_+β_6_InPopulation_it_+e_it_

(Paths are Construction, FDI, Export, Import. Control variables are resource moderation, corruption index, industrial added value and agricultural added value in the basic control variables.)

β is the estimated coefficient for measuring all variables, e_it_ is the error term, country and time are respectively subscripted by i (i = 1, 2, 3,…, N), t (i = 1, 2, 3,…, N).

### 3.4. Data test

#### 3.4.1 Description of statistics

As can be seen in Table 3 from descriptive statistics, Africa’s per capita carbon emission from 2003 to 2014 was 1.7197, much lower than China’s 5.6785. Among the different communication paths between China and Africa, foreign direct investment from China is the lowest, and Africa imports from China is the most. This suggests that Africa spends more on Chinese goods than it earns from Chinese exports and from Chinese foreign direct investment. It is worth noting that the minimum value for FDI from China is negative, suggesting that Chinese investment may be withdrawing. Finally, large standard deviation gaps indicate differences between sample countries.

**Table 3:** Descriptive statistics.

	Mean	Median	Maximum	Minimum	Std. Dev.
CO2	1.7197	0.7586	9.3839	0.0208	2.2628
CONSTRUCTION	690.9899	204.8500	7556.4000	0.9000	1303.2570
FDI	60.2318	8.6450	4807.8600	-814.9100	286.8965
EXPORT	1319.7960	176.1200	33561.9000	0.0000	4169.1000
IMPORT	1633.4050	590.1150	16830.7800	1.7800	2656.8100
ENERGY	825.3135	502.7945	3353.5260	9.5481	777.3499
GDPGROWTH	4.9659	5.0595	123.1396	-62.0759	8.8180
GDPPERCAPITA	2828.0220	1374.9820	14382.6000	119.4904	2978.3490
POPULATION	28162836	15325353	1.76E+08	82475	33851034

#### 3.4.2 Unit root test

Before the empirical analysis, in order to avoid pseudo-regression and ensure the validity of the estimated results, the stationarity of the panel series is tested [33].

LLC and IPS unit root tests are used in this paper to test the stationarity of the sequence. The unit root test results of panel data are shown in Table 4. According to LLC inspection, FDI, energy, GDP growth, GDP Percapita, Population, Industry, value added, Moderation—FDI, Corruption and Agriculture Value added was stable at the level. However, according to the IPS test, only FDI, energy, GDP growth, Population, moderation-fdi, Corruption and Agriculture had stable value added. However, after the first difference all variables are stationary.

**Table 4:** Unit root test.

Variables		At level		At 1st difference	
		Statistic	Prob.	Statistic	Prob
LC unit root test					
co2		-3.5549	0.0002	-13.0475	0.0000
Construction		4.0152	1.0000	-10.6040	0.0000
FDI		-4.4945	0.0000	-16.7822	0.0000
export		1.3734	0.9152	-6.3576	0.0000
import		1.7917	0.9634	-13.4559	0.0000
energy		-16.8596	0.0000	-10.8609	0.0000
GDP growth		-16.2099	0.0000	-23.0690	0.0000
GDP Percapita		-5.5500	0.0000	-15.3718	0.0000
Population		-6.7006	0.0000	-12.4597	0.0000
Industry, value added		-3.2724	0.0005	-24.1531	0.0000
moderation-construction		3.0771	0.9990	-10.7831	0.0000
moderation-fdi		-3.4407	0.0003	-1.7364	0.0412
moderation-export		0.4608	0.6775	-9.9281	0.0000
moderation-import		1.1048	0.8654	-11.9248	0.0000
Corruption		-7.5956	0.0000	-21.8491	0.0000
Agriculture, value added		-5.3824	0.0000	-16.9035	0.0000
IPS unit root test					
co2		-0.0987	0.4607	-8.2871	0.0000
Construction		7.1264	1.0000	-8.2706	0.0000
FDI		-2.7588	0.0029	-12.9951	0.0000
export		7.2665	1.0000	-5.8554	0.0000
import		6.6238	1.0000	-7.8886	0.0000
energy		-2.9106	0.0018	-7.3965	0.0000
GDP growth		-9.5618	0.0000	-16.3787	0.0000
GDP Percapita		-0.1101	0.4562	-9.6022	0.0000
Population		-19.6826	0.0000	-15.1007	0.0000
Industry, value added		-1.3740	0.0847	-10.6771	0.0000
moderation-construction		6.3888	1.0000	-8.8174	0.0000
moderation-fdi		-1.7364	0.0412	-11.8591	0.0000
moderation-export		4.3994	1.0000	-7.3850	0.0000
moderation-import		6.2826	1.0000	-6.7979	0.0000
Corruption		-2.4209	0.0077	-12.6144	0.0000
Agriculture, value added		-2.5188	0.0059	-10.2220	0.0000

#### 3.4.3 Cointegration test

In order to further study whether there is a long-term equilibrium relationship between the variables, we conducted a cointegration test on the variables, and we used the Pedroni method to test whether there is a cointegration relationship between the sequences Gulfer [34].

The results in Table 5 show that two thirds of the cases are significant, indicating that the null hypothesis without co-integration is rejected. Thus, carbon emissions, construction, foreign direct investment from China, African imports from China, African exports to China, energy consumption, GDP percapita, GDP growth and population are co-integrated in this panel.

**Table 5:** Cointegration test with basic control variables.

		Panel v-Statistic	Panel rho-Statistic	Panel PP-Statistic	Panel ADF-Statistic	Group rho-Statistic	Group PP-Statistic	Group ADF-Statistic
(1)	Statistic	-4.2410	5.2819	-6.2200	-2.8624	7.2690	-8.9499	-3.8754
	Prob.	1.0000	1.0000	0.0000	0.0021	1.0000	0.0000	0.0001
(2)	Statistic	-3.5487	4.6670	-5.7745	-3.4335	7.0693	-6.3414	-2.1148
	Prob.	0.9998	1.0000	0.0000	0.0003	1.0000	0.0000	0.0172
(3)	Statistic	-4.0761	4.9678	-7.4203	-3.7853	7.2572	-9.4060	-3.3728
	Prob.	1.0000	1.0000	0.0000	0.0001	1.0000	0.0000	0.0004
(4)	Statistic	-4.3561	4.8470	-5.7286	-3.1812	6.9340	-6.3675	-2.2238
	Prob.	1.0000	1.0000	0.0000	0.0007	1.0000	0.0000	0.0131

At the same time, the co-integration test was conducted in Tables 6–9 on the data of added natural factors, social factors, industrial factors and agricultural factors, and it can be seen that most of the results are significant, indicating that the data after adding various factors are also co-integrated.

**Table 6:** Cointegration test with natural factors added.

		Panel v-Statistic	Panel rho-Statistic	Panel PP-Statistic	Panel ADF-Statistic	Group rho-Statistic	Group PP-Statistic	Group ADF-Statistic
1	Statistic	-5.3833	6.7519	-7.5845	-2.2692	8.7551	-10.3215	-3.0082
	Prob.	1.0000	1.0000	0.0000	0.0116	1.0000	0.0000	0.0013
2	Statistic	-4.5267	6.3243	-5.6297	-2.2208	8.1786	-9.0126	-2.4935
	Prob.	1.0000	1.0000	0.0000	0.0132	1.0000	0.0000	0.0063
3	Statistic	-4.4958	5.9027	-9.4378	-4.7309	8.3461	-12.2099	-3.3031
	Prob.	1.0000	1.0000	0.0000	0.0000	1.0000	0.0000	0.0005
4	Statistic	-6.4262	7.8935	-11.103	-3.3934	9.7198	-15.1728	-2.3834
	Prob.	1.0000	1.0000	0.0000	0.0003	1.0000	0.0000	0.0086

**Table 7:** Co-integration test with social factors added.

		Panel v-Statistic	Panel rho-Statistic	Panel PP-Statistic	Panel ADF-Statistic	Group rho-Statistic	Group PP-Statistic	Group ADF-Statistic
1	Statistic	-4.5780	6.1241	-10.3927	-4.4631	8.4637	-14.1422	-4.2112
	Prob.	1.0000	1.0000	0.0000	0.0000	1.0000	0.0000	0.0000
2	Statistic	-4.3979	5.9727	-7.2835	-3.6218	8.1517	-12.8725	-3.9949
	Prob.	1.0000	1.0000	0.0000	0.0001	1.0000	0.0000	0.0000
3	Statistic	-4.3979	5.9727	-7.2835	-3.6218	8.1517	-12.8725	-3.9949
	Prob.	1.0000	1.0000	0.0000	0.0001	1.0000	0.0000	0.0000
4	Statistic	-5.1997	5.7808	-9.4870	-4.9185	8.2474	-12.5456	-2.9480
	Prob.	1.0000	1.0000	0.0000	0.0000	1.0000	0.0000	0.0016

**Table 8:** Co-integration test with industrial factors added.

		Panel v-Statistic	Panel rho-Statistic	Panel PP-Statistic	Panel ADF-Statistic	Group rho-Statistic	Group PP-Statistic	Group ADF-Statistic
(1)	Statistic	-4.5780	6.1241	-10.3927	-4.4631	8.4637	-14.1422	-4.2112
	Prob.	1.0000	1.0000	0.0000	0.0000	1.0000	0.0000	0.0000
(2)	Statistic	-4.3979	5.9727	-7.2835	-3.6218	8.1517	-12.8725	-3.9949
	Prob.	1.0000	1.0000	0.0000	0.0001	1.0000	0.0000	0.0000
(3)	Statistic	-4.3979	5.9727	-7.2835	-3.6218	8.1517	-12.8725	-3.9949
	Prob.	1.0000	1.0000	0.0000	0.0001	1.0000	0.0000	0.0000
(4)	Statistic	-5.1997	5.7808	-9.4870	-4.9185	8.2474	-12.5456	-2.9480
	Prob.	1.0000	1.0000	0.0000	0.0000	1.0000	0.0000	0.0016

**Table 9:** Co-integration test with agricultural factors added.

		Panel v-Statistic	Panel rho-Statistic	Panel PP-Statistic	Panel ADF-Statistic	Group rho-Statistic	Group PP-Statistic	Group ADF-Statistic
(1)	Statistic	-5.3820	6.4359	-9.8395	-3.6113	8.6674	-14.3394	-3.3722
	Prob.	1.0000	1.0000	0.0000	0.0002	1.0000	0.0000	0.0004
(2)	Statistic	-4.7109	5.9854	-8.1415	-4.0232	8.5140	-11.0260	-2.0329
	Prob.	1.0000	1.0000	0.0000	0.0000	1.0000	0.0000	0.0210
(3)	Statistic	-4.7819	6.0229	-9.5296	-4.4491	8.6875	-13.2571	-2.3130
	Prob.	1.0000	1.0000	0.0000	0.0000	1.0000	0.0000	0.0104
(4)	Statistic	-5.1649	6.3702	-7.1659	-2.8531	8.521	-9.7750	-1.8871
	Prob.	1.0000	1.0000	0.0000	0.0022	1.0000	0.0000	0.0296

## 4. Empirical results

### 4.1 Results under basic control variables

Based on the variable intercept model, we choose energy use, GDP growth, GDP per capita, and population as control variables, and the main results are shown in Table 10.

**Table 10:** Main results.

	Construction		FDI		Export		Import	
	(1)		(2)		(3)		(4)	
Variable	Coefficient	Prob.	Coefficient	Prob.	Coefficient	Prob.	Coefficient	Prob.
Construction	0.0003***	0.0044						
FDI			-0.000004**	0.0245				
Export					0.0002**	0.0218		
Import							-0.0003**	0.0164
energy	0.0041***	0.0000	0.0043***	0.0000	0.0043***	0.0000	0.0041***	0.0000
GDP growth	0.0061***	0.0000	0.0052***	0.0000	0.0059***	0.0000	0.0050***	0.0000
GDP Percapita	0.0004***	0.0181	0.0005***	0.0044	0.0006***	0.0007	0.0003*	0.0596
Population	0.0076	0.1510	0.0095*	0.0775	0.0102*	0.0536	0.0092*	0.0814

The results in column 1 show a positive relationship between building revenue and carbon emissions. Our explanation for this is that China’s construction activities in Africa generate carbon emissions. The white paper "China-Africa Cooperation in a New Era" shows that from 2000 to 2020, China has helped build more than 13,000 kilometers of roads and railroads in Africa, constructed more than 80 large-scale power facilities, and assisted in building more than 130 medical facilities, 45 stadiums, and more than 170 schools, all of which are set to increase CO2 emissions. Among the basic control variables, energy consumption, GDP growth, and GDP per capita are all significant at the 1% level, and the coefficients are all positive. According to the Kuznets curve, in the process of economic development, environmental conditions first deteriorate and then gradually improve. At present, Africa is an extensive economic development model. The use of resources will lead to increased carbon emissions and environmental degradation in Africa. With the growth of per capita GDP, the scale of the economy is getting larger and larger, and more resources need to be consumed to increase carbon emissions, thereby reducing the quality of the environment. Population is not significant in this path.

In the second column, we found that the FDI coefficient is negative. We generally believe that China’s investment in Africa is mainly used to develop natural resources, which will lead to environmental degradation in African countries, but the result is not. China’s aid to Africa is for natural resources is a widespread misunderstanding. China’s investment in Africa is more of a public diplomacy strategy to build friendly relations and win international support in the future. Over the past two decades, China-Africa investment cooperation has made great strides, with China’s new direct investment in Africa amounting to $4.2 billion in 2020, 56 times more than in 2003. China has been increasing its experience and technical assistance to Africa on the basis of what it can do to help Africa’s green transformation. In this perspective, the results of FDI support the pollution halo hypothesis that China’s direct investment in Africa is beneficial to Africa’s environment. Meanwhile, the coefficients of the four control variables are all positive. Indicating that energy use and economic development will increase carbon emissions in Africa. Population is significant at the 10% level, indicating that the increase in food demand, travel or housing demand caused by the increase of population leads to the increase in carbon emissions.

Column 3, The results show that African countries’ exports to China have a significant negative impact on the African environment, and we believe that domestic production and export will generate carbon emissions and have an adverse impact on the domestic environment. With the advancement of Africa’s industrialization process and the deepening of China-Africa exchanges, exports are bound to expand, and the expansion of production will also lead to increased carbon emissions and damage to the African environment. China has taken the initiative to expand imports of non-resource products from Africa, providing zero-tariff treatment for 97% of tariff lines of products from 33 African LDCs exported to China, helping more African agricultural and manufacturing products enter the Chinese market, assisting African economic development. Therefore, the results of export path support the pollution haven hypothesis. The significance of the control variables of African countries’ export paths to China is consistent with that of China’s foreign direct investment paths to Africa. From the comparison of coefficients, the coefficient of population is the largest in the path of Africa’s export to China, indicating that the negative impact of population on the environment is the largest in the path of Africa’s exchange with China.

In column 4, The coefficient -0.0003 shows that imports and carbon dioxide are negatively correlated at 5% level. We believe that Africa imports goods from China because the carbon emissions from the production of goods remain in China, so imports reduce Africa’s carbon emissions. The structure of China-Africa trade continues to be optimized, and the technological content of China’s exports to Africa has increased significantly, with the export value of electromechanical and high-tech products to Africa accounting for more than 50%. This path also supports the pollution halo hypothesis. Compared to the previous path, the effect of population on the environment remained the largest of these control variables.

### 4.2 Results of adding resource moderation

In this part, we examine whether natural resource factors have a significant impact on carbon emissions in China-Africa trade [1]. We introduced resource moderation into the model as a representative of natural factors, reflecting the interaction between different participation paths and natural resources.

Table 11 shows the results of adding the control variable " resource moderation ". In the first column, the relationship between the construction revenue path and carbon emissions after adding resource adjustment is no longer significant, that is to say, after controlling for natural resource factors, China’s construction revenue in Africa has no relationship with Africa’s carbon emissions. At the same time, the resource moderation as a control variable does not show significance in this path, indicating that there is no direct relationship between natural resources and the path of construction income. Moreover, the coefficients of the four basic variables are all positive. Among them, energy consumption, GDP growth and GDP per capita show significant, while population factor does not show significant. GDP growth has the biggest impact on the environment of Africa, while population has nothing to do with the environment of Africa.

**Table 11:** Results of adding resource moderation.

	Construction		FDI		Export		Import	
	(1)		(2)		(3)		(4)	
Variable	Coefficient	Prob.	Coefficient	Prob.	Coefficient	Prob.	Coefficient	Prob.
Construction	0.00161	0.4140						
FDI			-0.000002	0.8509				
Export					0.0002**	0.0395		
Import							-0.0010	0.6509
energy	0.0040***	0.0000	0.0042***	0.0000	0.0043***	0.0000	0.0041***	0.0000
GDP growth	0.0059***	0.0000	0.0058***	0.0000	0.0058***	0.0000	0.0051***	0.0000
GDP Percapita	0.0004**	0.0142	0.0006***	0.0008	0.0006***	0.0005	0.0004*	0.0633
Population	0.0070	0.1875	0.0101**	0.0565	0.0097*	0.0671	0.0090*	0.0933
Resource moderation	-0.0191	0.4996	-0.0032*	0.0250	-0.0021*	0.0551	-0.0095	0.7605

In the second column, after adding natural factors to the control variables, China’s direct investment in Africa is not significant. From the control variables, the resource adjustment of this path is significant at the 10% level with a negative coefficient. We believe that the reason why natural resources are beneficial to Africa in reducing carbon emissions is that it has abundant natural resources and is conducive to helping African countries to improve their energy structure. Increasing the proportion of renewable energy in the energy structure can effectively reduce carbon emissions. At the same time, abundant forest resources are used to absorb, collect, remove, fix and utilize carbon dioxide in the atmosphere, and return carbon dioxide to the biosphere, lithosphere, hydrosphere and pedosphere. The four basic control variables all show a high level of significance, and the coefficients are all positive. Population has the greatest impact on the environment in Africa.

In the third column, it can be found that African exports to China are the only significant path among the four paths, and after the adjustment of resources, it is positively correlated with carbon emissions. For the resource adjustment of the control variable, the resource adjustment of this path is significant at the 10% level, and its coefficient is negative, indicating that the more abundant the natural resources of this path, the more conducive to environmental protection in Africa. The reason is the same as that in the previous path. For the four basic control variables, the coefficients are all positive. Energy consumption, GDP growth and GDP per capita are significant at 1% level, and population is significant at 10% level. Comparing the coefficients of each basic variable, the coefficient of population is still the highest, indicating that population has the greatest impact on the environment in Africa after adding the resource adjustment.

The last column is the path of Africa’s imports from China. The path is not significant after adding resource adjustments. Resource restraint makes no sense on this path. The significance of the four basic control variables has some changes compared to the first three paths. Energy consumption and GDP growth are significant at the 1% level, and per capita GDP and population are significant at the 10% level, with population still having the greatest impact on the African environment.

### 4.3 Results of adding corruption index

The previous section discussed the impact of natural factors on carbon emissions, but the results were not significant. According to Biswas et al. [35] and Alessandra et al. [36] studies, corruption indirectly affects the environment through other factors or loopholes in environmental regulations. Therefore, we choose the corruption index as a control variable to add to the basic control variables.

In the first column of Table 12, after adding the corruption index as a control variable, the construction revenue is significant at the 1% level, and the coefficient is positive. However, the control variable of the corruption index is not significant on this path, indicating that the corruption index has no impact on the African environment on the construction income path. For the basic control variables, energy consumption and GDP growth are significant at the 1% level with positive coefficients. GDP per capita is significant at the level of 10%, while population is not significant.

**Table 12:** Results of adding corruption index.

	Construction		FDI		Export		Import	
	(1)		(2)		(3)		(4)	
Variable	Coefficient	Prob.	Coefficient	Prob.	Coefficient	Prob.	Coefficient	Prob.
Construction	0.0003***	0.0038						
FDI			-0.000005*	0.1033				
Export					0.0002**	0.0170		
Import							-0.0003**	0.0163
energy	0.0041***	0.0000	0.0043***	0.0000	0.0044***	0.0000	0.0041***	0.0000
GDP growth	0.0062***	0.0000	0.0053***	0.0023	0.0061***	0.0000	0.0051***	0.0000
GDP Percapita	0.0004*	0.0401	0.0005***	0.0006	0.0006***	0.0022	0.0003*	0.0832
Population	0.0066	0.2622	0.0088*	0.0750	0.0102**	0.0839	0.0082	0.1574
Corruption	0.0023	0.6378	0.0010***	0.0005	0.0037	0.4652	0.0008	0.8759

In the second column, China’s foreign direct investment is still negative after adding the corruption index, indicating that China’s foreign direct investment is conducive to reducing carbon emissions in Africa. Corruption index is significant and the coefficient is positive, indicating that corruption indirectly has a negative impact on the environment through other factors or loopholes in environmental regulations. We believe that if a government is corrupt, it will focus more on short-term economic benefits than long-term environmental impacts. Second, corruption can lead to the deterioration and ineffectiveness of environmental policies, which can negatively impact the environment. Basic control variables also show importance in this path. After adding the corruption index, the significance of the underlying control variables changed compared to the previous path. The per capita GDP has increased significantly, and the population has also increased significantly at the 10% level. This shows that in China’s FDI to Africa, the degree of environmental impact of per capita GDP and population growth has increased.

In the third column, Africa’s export to China has a positive result coefficient at 5% level. The corruption index does not show significant in this path, indicating that the corruption problem in the export path of Africa to China will not have impact on Environment of Africa. The remaining four control variables are showing a significant positive correlation, and population has the greatest impact on the environment.

In the fourth column, after adding the corruption index as a control variable, Africa’s import path from China shows a significant negative correlation. As a control variable, the corruption index does not show significant in this path. The population factor was not significant as the basic control variable. The control variables energy consumption, GDP growth and GDP per capita show significant positive correlation in the path table of Africa’s imports from China with the corruption index. GDP growth has the biggest impact on the environment.

### 4.4 Results of adding industrial added value

This part uses industrial added value as a control variable and discusses the impact of multiple paths between China and Africa on the African environment after controlling for industrial factors. Previous studies by scholars Wang [37], Li [38] believes that the cooperation between China and Africa on industrialization can greatly promote the industrialization development of Africa, but it will cause certain damage to the environment of Africa.

In the first column of Table 13, after adding the industrial added value, all variables in Path 1 except the population factor are significant and the coefficients are all positive. African countries are in the stage of rapid industrial development. On the one hand, the rapid development of the industry needs the support of energy consumption. On the other hand, the industrial production process will also produce pollution emissions. After increasing the industrial added value, the construction revenue path is still significant, which supports the pollution shelter hypothesis. The last is four basic variables, and population factor does not show significant in this path. GDP growth has the greatest negative impact on the African environment.

**Table 13:** Results of adding industrial factors.

	Construction		FDI		Export		Import	
	(1)		(2)		(3)		(4)	
Variable	Coefficient	Prob.	Coefficient	Prob.	Coefficient	Prob.	Coefficient	Prob.
Construction	0.0003***	0.0060						
FDI			-0.0000025**	0.0364				
Export					0.0002**	0.0172		
Import							-0.0002*	0.0623
energy	0.0040***	0.0000	0.0042***	0.0000	0.0042***	0.0000	0.0041***	0.0000
GDP growth	0.0049***	0.0000	0.0040***	0.0001	0.0046***	0.0000	0.0039***	0.0001
GDP Percapita	0.0004**	0.0339	0.0005***	0.0097	0.0005***	0.0016	0.0003*	0.0678
Population	0.0063	0.2821	0.0082	0.1612	0.0093	0.1112	0.0080	0.1650
Industry	0.01427***	0.0001	0.0146***	0.0001	0.0145***	0.0001	0.0137***	0.0003

In the second column, all the factors are significant except the population factor. After adding the factor of industrial added value, the coefficient of China’s foreign direct investment in Africa is negative, indicating that the increase of China’s foreign direct investment in Africa is beneficial to the African environment, supporting the pollution halo hypothesis. The significance of industrial added value in this path are consistent with that in the previous path, and the coefficient has increase, indicating that the impact of industry on the environment in this path is more significant than that in the previous path. In this path, the coefficient of energy consumption of the basic control variable is the largest, which is inconsistent with most cases mentioned above. Energy consumption in this path has the biggest impact on carbon emissions in Africa.

In the third column of Africa’s export path to China, the significance levels of all variables in this path are consistent with the previous path, and the coefficients are all positive, indicating that this path supports the pollution haven hypothesis.

Finally, the fourth column is Africa’s import path from China. After adding the factor of industrial added value, Africa’s imports from China have a negative correlation with the African environment. The significance of industrial added value under this path is still high, but the coefficient has decreased compared with the previous paths, indicating that the impact of industrial added value under this path on the African environment has decreased. The population factor in the basic control variables is still insignificant, the significance of GDP per capita is reduced, and its coefficient is also reduced compared with other paths.

### 4.5 Results of adding agricultural added value

Since the beginning of the 21st century, under the influence of the successful experience of other developing countries, the African continent has begun to rearrange its agricultural development, so as to get rid of the situation that African agriculture depends on other countries and embark on the road of agricultural development. Independently develop agriculture.

According to Li et al. [39], the interaction of agricultural development in Africa with climate and environmental change. In this section, we choose agricultural value added as a control variable to test whether agricultural factors in China-Africa exchanges have a significant impact on the environment.

In the first column of Table 14, the construction income path after adding agricultural value-added shows that the population factor is not significant, and the agricultural value-added factor in the path is not significant, indicating that the population and agricultural value-added, respectively, have nothing to do with carbon emissions in Africa.

**Table 14:** Results of adding agricultural added value.

	Construction		FDI		Export		Import	
	(1)		(2)		(3)		(4)	
Variable	Coefficient	Prob.	Coefficient	Prob.	Coefficient	Prob.	Coefficient	Prob.
Construction	0.0003***	0.0036						
FDI			-0.000004**	0.0329				
Export					0.0002**	0.0154		
Import							-0.0003**	0.0178
energy	0.0041***	0.0000	0.0043***	0.0000	0.0043***	0.0000	0.0041***	0.0000
GDP growth	0.0061***	0.0000	0.0052***	0.0000	0.0059***	0.0000	0.0050***	0.0000
GDP Percapita	0.0004***	0.0012	0.0005***	0.0047	0.0006***	0.0006	0.0004*	0.0539
Population	0.0073	0.1418	0.0093*	0.0871	0.0102*	0.0593	0.0090*	0.0941
Agriculture	0.0012	0.7600	0.0006	0.9446	0.0005	0.9473	0.0012	0.8769

The second column is the path of China’s foreign direct investment in Africa after adding agricultural value-added factors. Comparing the results in Tables 10 and 14, it can be found that the significance and coefficient of China’s OFDI in Africa and the basic control variables of energy consumption, GDP growth and per capita GDP are the same. The significance of the population factor is the same, and the coefficient increases, while the impact of other factors on the environment is the same as that without adding the agricultural value-added factor. In Table 14, agricultural value added is not significant along this path.

The third column is the export path of Africa to China, and the agricultural value added of this path is not significant. Other variables: African exports to China, energy consumption, GDP growth and per capita GDP population, compared with the results in Table 10, the coefficients and significance are the same, and the agricultural added value does not increase, indicating the impact of each variable on the African environment. The same path as without increasing agricultural value added.

And Africa imports from China. The added agricultural value added in this path does not show significant.

### 4.6 Discussion

This paper interprets the regression results of the four paths of China-Africa exchanges: construction, FDI, exports and imports on Africa’s environmental impacts, and adds four basic control variables. Use natural factors, social factors, industrial factors, and agricultural factors to improve the content covered by the basic control variables, and more comprehensively reflect the impact of various paths of China-Africa exchanges on the African environment. Based on the above interpretation of the results, the following findings are made:

1. Building revenue is positively correlated with carbon emissions. Chinese construction projects in Africa increase carbon dioxide emissions. This result supports the pollution haven hypothesis.
2. In contrast to construction revenue, the coefficient of Chinese OFDI is negative, suggesting that Chinese OFDI to Africa is associated with a reduction in CO2 emissions. This result supports the pollution halo hypothesis.
3. Africa’s import and export to China and carbon emission coefficients are both positive, supporting the pollution haven hypothesis. African imports from China are negatively correlated.

Incorporating natural and social factors into the above four pathways, and exploring the direct relationship between these two factors and carbon emissions on the above four pathways, our results are different from Tawiah et al. [1], We believe that natural factors have no impact on the carbon emissions of each path. On this basis, we also added industrial factors and agricultural factors. We found that agricultural factors had no effect on carbon emissions, and industrial factors increased carbon emissions.

There is also economic reasoning behind the study’s findings; construction revenues are positively correlated with carbon emissions in Africa: this means that as construction revenues increase, carbon emissions in Africa also increase. Construction activities in Africa typically require significant use of energy and materials, leading to an increase in carbon emissions. As Africa’s economy grows, the construction industry develops rapidly and the number of construction projects increases, which leads to an increase in carbon emissions.

Chinese OFDI in Africa can reduce CO2 emissions in Africa: Chinese investment in Africa has helped drive economic development in African countries, gradually shifting them from traditional high-carbon-emitting industries to low-carbon economies and reducing carbon emissions. China has increased its investment in clean energy in Africa, including renewable energy projects such as solar and wind energy. At the same time, China can help African countries develop clean energy industries and reduce their reliance on traditional high-carbon energy sources through technology transfer and training cooperation.

Africa’s imports and exports to China are positively correlated with Africa’s carbon emissions: this means that Africa’s imports from China are associated with higher carbon emissions, and the import structure of African countries may tend to favor carbon-intensive products, such as energy and industrial goods, leading to an increase in carbon emissions. African countries can strengthen the construction of environmental standards and certification, and provide technical guidance and support to enterprises to ensure that the products produced and imported meet environmental and low-carbon requirements. African countries export resource-based products, such as oil and minerals, to China. These resource-based products are usually associated with high carbon emissions.

## 5. Conclusions

The growing trade exchanges between China and Africa have played a role in promoting the development of African countries. However, the problems exposed during the deepening of trade exchanges have also aroused people’s concerns about the environmental problems of African continent. On the one hand, China may bring its environmentally unfavorable activities to Africa, causing African carbon emissions to increase. On the other hand, China has reduced its carbon emissions in China-Africa trade by increasing investment in renewable energy and other clean energy in African countries.

The results of our research are as follows. China’s construction activities have increased Africa’s carbon emissions, and Africa’s exports to China will also increase carbon emissions. Africa’s imports from China and China’s foreign direct investment in Africa will reduce the carbon emissions of African countries. As for the control variables: energy consumption, GDP growth, GDP per capita, and population, all show a positive correlation with Africa’s carbon emissions under various paths. We add various influencing factors as control variables: natural resource adjustment, corruption index, industrial value-added and agricultural value-added, which show different significance in the various paths of China-Africa exchanges.

Our results show that each path of China’s contact with Africa may have different impact on African environment. Therefore, we make the following suggestions for the exchanges between China and Africa.

First, in construction and export activities, Chinese government and companies actively encourage increased construction and export activities between China and Africa. Although construction activities combined with export activities will increase Africa’s carbon emissions at this stage, in the long run, Africa’s development needs China’s support in construction and trade. China can further assist Africa in construction, technological upgrading and economic structural transformation. China and Africa should coordinate the contradiction between economic development and carbon emission reduction, so that development and environmental protection can develop together.

Second, in terms of direct investment and to imports, Africa needs to pay attention to the role of Chinese investment and imports from China in Africa’s economic development and carbon emission reduction. Africa should direct investment to improve the energy mix, increase the use of renewable energy and structural transformation of the economy, and import more products and services that are high in energy consumption and pollution. Improve the utilization efficiency of foreign direct investment and imported goods and services from China to better reduce CO2 emissions Third, the environmental protection strategy of China-Africa cooperation should be adapted to local conditions. When formulating China-Africa environmental policies, it is necessary to fully understand the needs of African countries, especially the infrastructure construction needs of African countries in environmental protection. Only with targeted environmental protection policies can we develop more effectively and sustainably.

This paper hopes that by studying the impact of China-Africa exchanges on the African environment, it can serve as a reference for other countries’ exchanges on the environment, and also hope to help other countries or regions with similar situations to use our research results to formulate reasonable policies. The shortcoming of this paper is the lack of research on the environmental impact of political factors in Sino-African exchanges, which needs to be supplemented by scholars in the future.
